# Ubenimex induces autophagy inhibition and EMT suppression to overcome cisplatin resistance in GC cells by perturbing the CD13/EMP3/PI3K/AKT/NF-κB axis

**DOI:** 10.18632/aging.102598

**Published:** 2019-12-31

**Authors:** Qie Guo, Fan-Jing Jing, Wen Xu, Xiao Li, Xin Li, Jia-Lin Sun, Xiao-Min Xing, Chang-Kai Zhou, Fan-Bo Jing

**Affiliations:** 1 Department of Clinical Pharmacy, The Affiliated Hospital of Qingdao University, Qingdao, Shandong 266003, PR China; 2 Department of Oncology, The Affiliated Hospital of Qingdao University, Qingdao, Shandong 266003, PR China

**Keywords:** Ubenimex, CD13, EMP3, EMT, autophagy

## Abstract

Cisplatin (CDDP)-based chemotherapy is a standard treatment for gastric cancer (GC). However, chemoresistance is a major obstacle for CDDP application. Exploring underlying mechanisms of CDDP resistance development in GC and selecting an effective strategy to overcome CDDP resistance remain a challenge. Here, we demonstrate that a transmembrane ectoenzyme, CD13, endows GC patients with insensitivity to CDDP and predicts an undesirable prognosis in GC patients with CDDP treatment. Similarly, CD13 expression is positively related with CDDP resistance in GC cells. A CD13 inhibitor, Ubenimex, reverses CDDP resistance and renders GC cells sensitivity to CDDP, for which CD13 reduction is essential, and epithelial membrane protein 3 (EMP3) is a putative target downstream of CD13. Furthermore, Ubenimex decreases EMP3 expression by boosting its CpG island hypermethylation for which CD13 down-regulation is required. In addition, EMP3 is a presumptive modifier by which CD13 exerts functions in the phosphoinositol 3-kinase/protein kinase B (PI3K/AKT) pathway. Ubenimex inhibits the activation of the CD13/EMP3/PI3K/AKT/NF-κB pathway to overcome CDDP resistance in GC cells by suppressing autophagy and epithelial-mesenchymal transition (EMT). Therefore, CD13 is a potential indicator of CDDP resistance formation, and Ubenimex may serve as a potent candidate for reversing CDDP resistance in GC.

## INTRODUCTION

Gastric cancer (GC) is the second leading cause of cancer-related mortality worldwide, with high morbidity and high-grade malignancy [1]. Although surgical removal is a curative treatment, systemic chemotherapy is an optimal therapeutic strategy for GC patients who are diagnosed at an advanced stage and consequently have distant metastases and poor prognosis, offering better outcomes than surgery alone [2, 3]. In particular, cisplatin (CDDP)-based chemotherapy has been approved as a treatment option for gastrointestinal cancers [4]. Unfortunately, in GC patients with CDDP treatment, tumor metastasis and local recurrence become increasingly common due to chemoresistance [5]. Emerging evidences have confirmed that autophagy induction [6, 7], the progress of epithelial-mesenchymal transition (EMT) [8, 9], and the overactivation of the phosphoinositol 3-kinase/protein kinase B (PI3K/AKT) pathway contribute to the development of CDDP resistance in GC [10, 11].

Epithelial membrane protein 3 (EMP3) belongs to the peripheral myelin protein 22-kDa (PMP22) gene family of small hydrophobic membrane glycoproteins [12]. EMP3 was reported to be a tumor suppressor gene and it underwent hypermethylation-mediated transcriptional silencing in Glioma, Esophageal squamous cell carcinoma (ESCC) and Non-small cell lung cancer (NSCLC) [13]. In contrast, some controversial data support that EMP3 acts as a novel marker of tumor aggressiveness showing upregulated mRNA expression and in gastric cancer-derived cell lines [14]. However, the roles of EMP3 in GC malignancy and its implications for GC therapy remain unclear to date.

CD13 is a transmembrane glycoprotein with metalloproteinase activity, and it encourages tumor angiogenesis and adhesion [15]. Also, CD13 is overexpressed in GC cells, of which protein levels are coincidentally correlated with the ability of cell invasion [16]. However, Ubenimex, a CD13 inhibitor, has only been used as an immuno-modulating adjuvant for treating hematological malignancies [17]. There is no report on the association between CD13 expression and CDDP resistance in GC cells, much less for the reversal of CDDP resistance mediated by Ubenimex.

In this study, we show that CD13 up-regulation is closely correlated with poor responses to CDDP and unsatisfactory overall survival in GC patients with CDDP-based chemotherapy. Exogenous CD13-expression wrecks GC cells sensitivity to CDDP, and CD13 is frequently expressed in CDDP-resistant GC cells, compared to their parental cells. CD13 knockdown significantly reduced the IC50 values and resistance index (RI) of CDDP-resistant GC cells towards CDDP. Pharmacological inhibition of CD13 by Ubenimex overcame CDDP resistance and enhanced GC cell sensitivity to CDDP. Further data confirmed that CD13 cause EMP3 upregulation unilaterally by restraining the CpG island hypermethylation in EMP3 DNA, and Ubenimex awaken the hypermethylation to down-regulate EMP3 expression, for which CD13 reduction is essential. We also established a molecular link between CD13 and the PI3K/AKT pathway, in which EMP3 was a positive regulatory pivot. Additionally, our findings affirmed that Ubenimex weakens the activation of the CD13/EMP3/ PI3K/AKT/NF-κB pathway to reverse CDDP resistance in GC, in which cell autophagy was inhibited, followed by an increase in CDDP-induced apoptosis, and EMT development was impeded, which was accompanied by the abatement of cell migration and invasion.

Altogether, this study not only suggests that CD13 is a promising target to reverse CDDP resistance in GC cells, but it also indicates that Ubenimex may be a feasible strategy for the treatment of GC.

## RESULTS

### Aberrant expression of CD13 is associated with poor CDDP sensitivity and prognosis of GC patients with CDDP treatment

**Figure 1:**
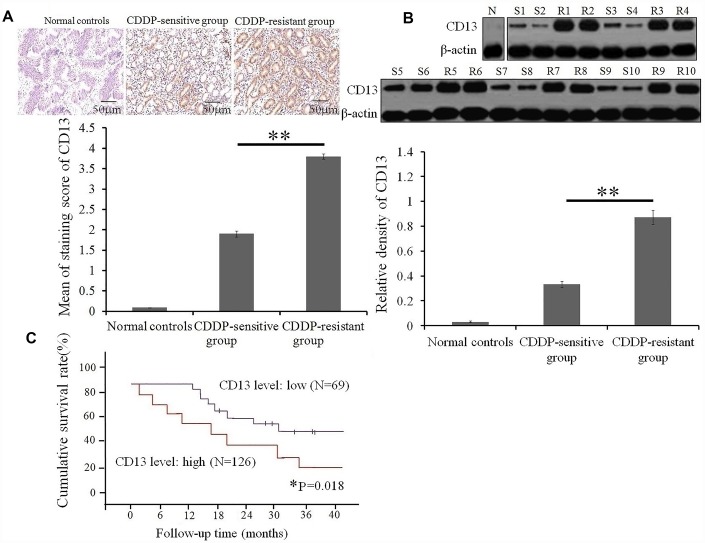
**High expression of CD 13 is a risk stratification strategy for GC patients with CDDP treatment.** (**A**) CD13 expression in serial sections from GC patients with CDDP-based chemotherapy was determined by *immunohistochemical staining.* Data are shown as the representative images (upper panels) and the staining score of CD13 with the means±SD (bottom panel) from three independent experiments. **P<0.01. (**B**) Comparison of CD13 expression between CDDP-sensitive and CDDP-resistant GC patients was conducted by Western blot assay. Data are displayed as the representatives (upper panels), and relative expression with means±SD (bottom panel) from three independent experiments.** P< 0.01. “S” and “R” represented the CDDP-sensitive and CDDP-resistant group, respectively. (**C**) The overall survival curves of GC patients based on the CD13 expression were generated using the Kaplan-Meier method. *P=0.018 was obtained using a log-rank test, and was considered to be statistically significant.

Up to now, the clinical relevance of CD13 expression in GC patients undergoing CDDP treatment remains unclear. Herein, we collected tumor specimens from 195 GC patients with CDDP-based chemotherapy, of which CD13 expression were assessed. No correlation between CD13 expression and clinicopathological factors, such as sex, age, clinical stage, and degree of differentiation, was observed (Table 1). IHC and Western blot assays both verified that CD13 expression was up-regulated in the CDDP-resistant group, compared to the CDDP-sensitive group (Figure 1A and 1B). Furthermore, as depicted in Figure 1C, GC patients exhibiting elevated CD13 levels had shorter overall survival (OS) time than those of the remaining parts.

**Table 1:** CD13 expression and the Clinico-pathological features of GC patients.

**Clinico-pathological features**	**CD13 expression**				
	**N**	**+**	**++**	**+++**	**P value**
Age					0.45
≤55years	97	34	30	33	
>55 years	98	35	37	26	
Gender					0.67
Male	91	29	34	28	
Female	104	40	33	31	
Depth of invasion					0.59
T_is-1_	91	33	32	26	
T_2-4_	104	36	35	33	
T Staging					0.49
T_1_- T_2_	91	31	30	30	
T_3_- T_4_	104	38	37	29	
N Staging					0.61
N0	107	39	36	32	
N_1_-N_3_	88	30	31	27	
Metastasis Staging					0.52
M_1_	101	36	30	35	
M_0_	94	33	37	24	
AJCC Staging					0.55
I-II	93	38	32	23	
III-IV	102	31	35	36	
Tumor Differentiation					0.76
Well	72	27	25	20	
Moderate	65	22	22	21	
Poor	58	20	20	18	

T_is_; cancer in situ; T_1_; invasion into lamina propria and submucosa; T_2_; invasion into muscularis propria and subserosa; T_3_; invasion into to serosa; T_4_; invasion into serosa, χ^2^ test were adopted to evaluate clinical parameters that were associated with CD13 expression. #P >0.05.

These findings suggest that CD13 may be potentially utilized as a chemotherapeutic response indicator and a prognostic biomarker in GC patients with CDDP-based chemotherapy.

### CD13 upregulation is correlated with development of CDDP resistance in GC cells

**Figure 2:**
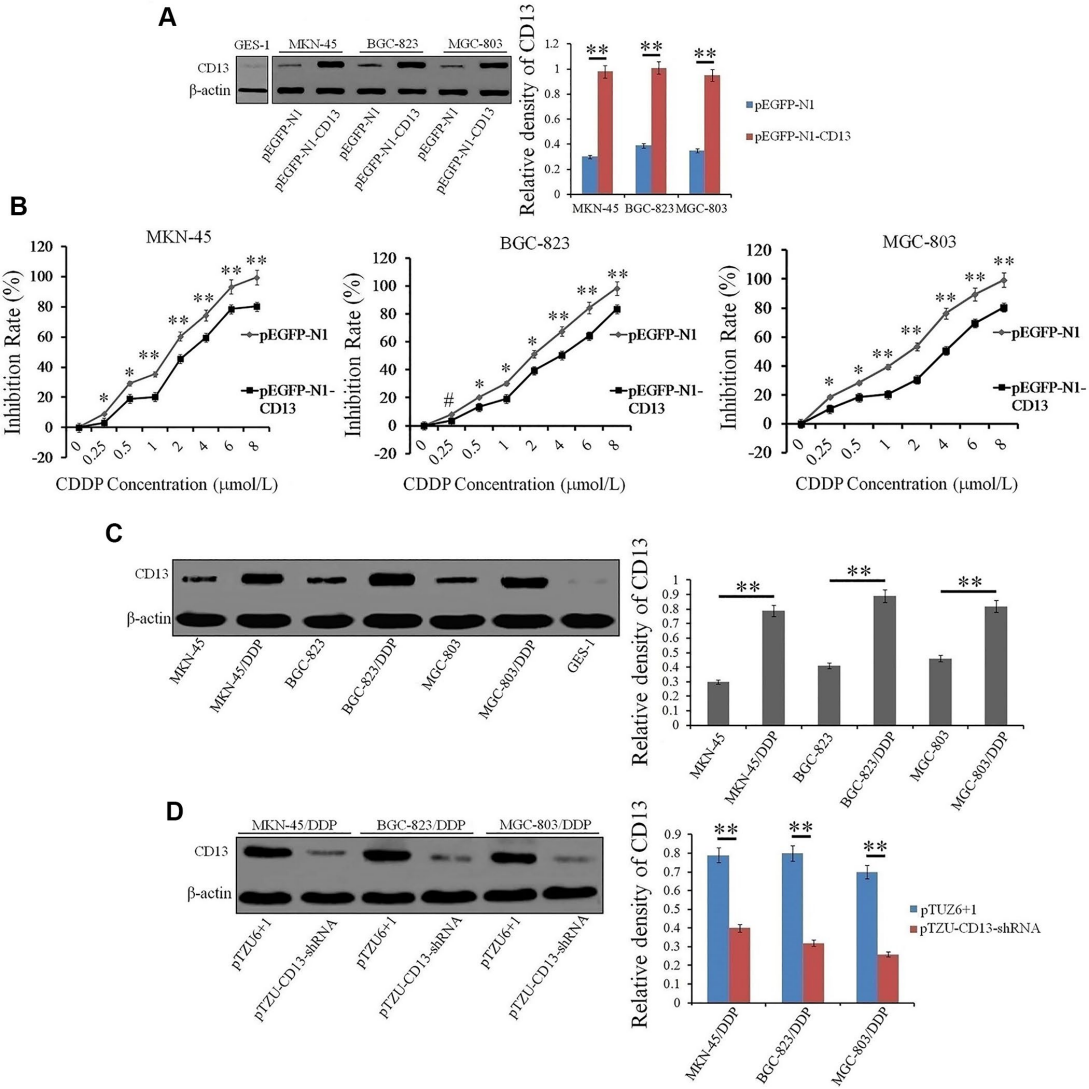
**CD13 expression is positively induced in CDDP-resistant GC cells compared to parental GC cells.** (**A**) Western blot assay was executed to evaluate the CD13 expression in GC cells that were transfected with exogenous CD13-expressing or control plasmids. (**B**) GC cells were transfected with indicated plasmids for 24h, and then treated with CDDP at increasing concentrations (0, 0.25, 0.5, 1, 2, 4, 6 and 8μmol/L) for another 48 h. Inhibitory effect of CDDP on the cell growth was determined by CCK-8 method. The results are shown as the means±SD of three independent experiments.*P<0.05, **P <0.01 and ^#^P>0.05. (**C**) Compared expression of CD13 in parental and CDDP-resistant GC cells was examined by Western blot assay. (**D**) Western blot assay was used to assess the CD13 expression in CDDP-resistant GC cells that were transfected with indicated plasmids. For Western blot assay, data are represented as the representatives (left panels) and relative expression with means±SD (right panels) from three independent experiments.**P<0.01.

Previously, CD13 has been shown to participate in maintaining stem cell characteristics and induce the chemoresistance of hepatoma carcinoma cells to 5-FU and Doxorubicin [18]. Thus, we hypothesized that CD13 overexpression leads to the deterioration of survival duration, in part by promoting CDDP resistance. Accordingly, GC cells were transfected with exogenous CD13-expressing plasmid pEGFP-N1-CD13, as described in Figure 2A and 2B, these GC cells have a greater abundance of CD13 protein, but become less sensitive towards CDDP after CD13 was over-expressed. We also prepared CDDP-resistant GC cell lines, and further confirmed that CDDP-resistant GC cells exhibit upregulated expression of CD13 compared to their parental cells (Figure 2C). Furthermore, pTZU-CD13-shRNA transfection did not only down-regulated CD13 expression (Figure 2D and Supplementary Figure 1), but also evidently reduced the IC50 values and RIs of CDDP-resistant GC cells towards CDDP (Table 2). These results bright to a reasonably positive correlation between CD13 expression and CDDP resistance in GC cells.

**Table 2:** The effect of CD13 knockdown on the IC50 values and RIs for CDDP-resistant GC and their parental cells to CDDP.

**Cell lines**	**IC50 (ug/ml)**		**RIs**	
	**pTZU6+1**	**pTZU-CD13-shRNA**	**pTZU6+1**	**pTZU-CD13-shRNA**
MKN-45	0.40±0.01	0.41±0.01	1	1
MKN-45/DDP	6.34±0.02	1.51±0.21**	15.82±0.13	3.75±0.19**
BGC823	0.55±0.01	0.57±0.01	1	1
BGC823/DDP	7.43±0.92	3.01±0.21**	12.89±0.23	5.60±0.19**
MGC-803	0.72±0.08	0.73±0.08	1	1
MGC-803/DDP	8.30±0.10	2.06±0.11**	13.91±0.23	2.85±0.12**

Indicated GC cells were pre-transfected with indicated plasmids for 24h, and then stimulated with CDDP (0, 2, 4, 8, and 16 μg/mL) for another 48 h. IC50 values and RIs were determined by the CCK-8 method. Data are expressed as the means±SD from three independent experiments. **P<0.01.

### Ubenimex abolishes CDDP resistance in GC cells by downregulating CD13 expression *in vitro*

**Figure 3:**
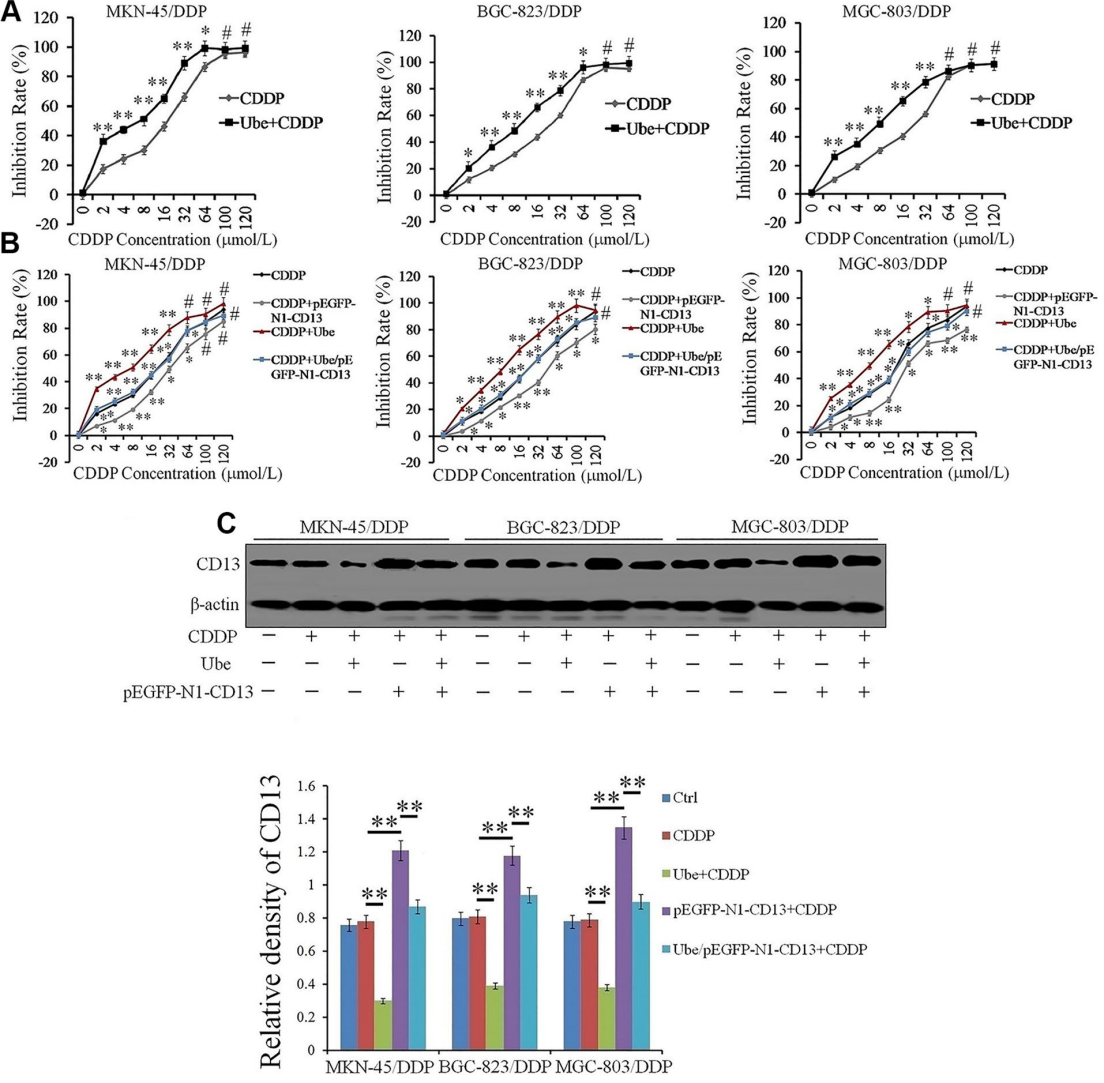
**Ubenimex reverses CDDP resistance in vitro by suppressing CD13 expression.** (**A**, **B**) CDDP-resistant GC cells were pre-treated with Ubenimex (0.2 mg/mL) and pEGFP-N1-CD13 or the combination of both for 24 h, followed by incubation with CDDP at increasing concentrations (0, 2, 4, 8, 16, 32, 64, 100, and 120 μmol/L) for another 48 h. CDDP cytotoxicity towards the indicated cells was manifested as the inhibition rate which was determined by CCK-8 method. The results are expressed as the means±SD of three replicates.*P <0.05, **P<0.01 and #P>0.05 versus CDDP and Ube/CDDP group. (**C**) CDDP-resistant GC cells were pre-stimulated with Ubenimex (0.2 mg/mL) and pEGFP-N1-CD13 or the combination of both for 24 h, and then treated with CDDP (20 μmol/L) for another 48h. Western blot assay was performed to determine CD13 expression. The representatives results (upper panels) and the means±SD of the relative expression (bottom panel) from three independent experiments were both demonstrated. **P< 0.01.

The observed correlation between CD13 expression and CDDP resistance prompted us to explore the effects of Ubenimex on CDDP treatment. As shown in Table 3, Ubenimex significantly reduced the IC50 values and RIs, and also markedly improved CDDP-resistant GC cell sensitivity to CDDP, but it had almost no impact on the proliferation of CDDP-resistant GC cells (Figure 3A and Supplementary Figure 2). However, no significant changes in cytotoxic activity of CDDP towards these parental cells with Ubenimex treatment were observed (Supplementary Figure 3). These findings suggest a threshold of CD13 expression for Ubenimex function, which is supported in some reports that pharmacological inhibition cannot be enabled when CD13 is lowly or moderately expressed [19]. Most interestingly, Ubenimex cannot render CDDP-resistant GC cells sensitivity to CDDP after CD13 was over-expressed (Figure 3B). Data achieved from Western Blot and quantitative Real-time PCR assays further confirmed that Ubenimex dramatically decreased CD13 expression in CDDP-resistant GC cells (Figure 3C and Supplementary Figure 4). In summary, Ubenimex reversed CDDP resistance and enhanced GC cells chemo-sensitivity to CDDP, of which the effect was largely dependent on the downregulation of CD13 expression.

**Table 3:** The effect of Ubenimex on the IC50 values and RIs for CDDP-resistant GC and their parental cells to CDDP.

**Cell lines**	**IC50 (ug/ml)**		**RIs**	
	**Control**	**Ubenimex**	**Control**	**Ubenimex**
MKN-45	0.40±0.01	0.40±0.02	1	1
MKN-45/DDP	6.37±0.03	1.91±0.04**	16.02±0.14	4.55±0.17**
BGC823	0.53±0.02	0.55±0.02	1	1
BGC823/DDP	7.63±0.60	2.91±0.11**	13.36±0.13	5.29±0.28**
MGC-803	0.72±0.08	0.73±0.06	1	1
MGC-803/DDP	8.36±0.09	2.14±0.09**	13.43±0.12	2.90±0.14**

Indicated GC cells were pre-treated with Ubenimex (0.2 mg/mL) for 24 h, and then stimulated with CDDP (0, 2, 4, 8, and 16 μg/mL) for another 48 h. IC50 values and RIs were determined by the CCK-8 method. Data are expressed as the means±SD from three independent experiments. **P<0.01.

### Ubenimex enhances CDDP efficacy against cell-based xenografts in vivo for which reduced expression of CD13 is essential

**Figure 4:**
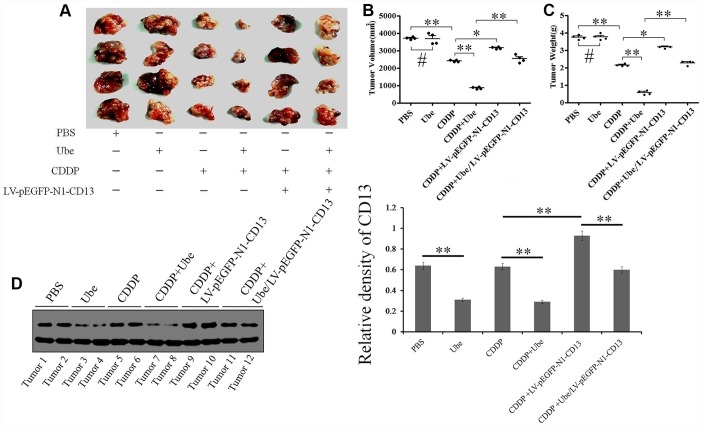
**Ubenimex enhances CDDP efficacy in cell-based xenografts by inhibiting CD13 expression.** (**A**) Tumor presentations in killed mice from each group were demonstrated. (**B**, **C**) The tumor volume (**B**) and tumor weight (**C**) with the means±SD from three independent experiments were evaluated. *P<0.05,**P<0.01 and #P>0.05 versus PBS, CDDP and Ube/CDDP group. (**D**) CD13 expression in tumor specimens of each group was assessed by Western blot assay. The results are demonstrated as the representatives (left panels) and the relative expression with the means±SD (right panel) from three irrelevant experiments. **P<0.01 versus PBS, CDDP and Ube/CDDP group.

According to the observation, we are intended to validate that whether Ubenimex can preferentially confer GC cells sensitivity to CDDP *in vivo*. Interestingly, the combination group treated with CDDP and Ubenimex exhibited a larger reduction in tumor burden (Figure 4A), and a more aggressive decrease in tumor volume and weight, compared to the single CDDP-treatment group (Figure 4B and 4C). However, enhancement in CDDP-sensitivity, and the pattern of changes in tumor volume and weight that were mediated by Ubenimex were abolished after CD13 was over-expressed via infection of LV-pEGFP-N1-CD13 plasmid (Figure 4A–4C). Furthermore, CD13 expression was substantially decreased in tumor sections from the single Ubenimex-treated and the combination group, compared with that from the control and single CDDP-treated group (Figure 4D). Ubenimex also reversed CDDP resistance and elevated expression of CD13 in the CD13 over-expressing group (Figure 4). Therefore, these results suggested that CD13 reduction contributes to the enhancement of CDDP-sensitivity induced by Ubenimex *in vivo*.

### EMP3 is a potential target of CD13 in CDDP-resistant GC cells with Ubenimex treatment

**Figure 5:**
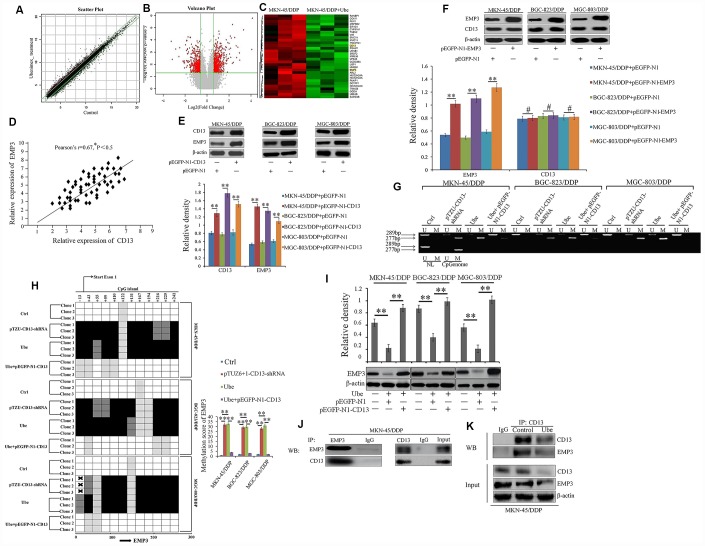
**EMP3 is a potential downstream target of CD13 in Ubenimex-treated CDDP-resistant GC cells.** (**A**) The scatter plot image showing the distribution of signal intensity in a rectangular coordinate plane. Red and green dots outside the interval represented the up-regulated probes in the Ubenimex-treated and control cells, respectively. (**B**) The volcano plot image for all of the genes. Gray and Red puncta represented equally (Fold Change<1.5) and differentially expressed mRNAs (Fold Change ≥1.5) between Ubenimex-treated MKN-45/DDP and MKN-45/DDP cells. (**C**) Heat map was generated using R package to depict 1306 transcripts that were significantly differentially expressed in MKN-45/DDP cells after Ubenimex treatment (Fold Change≥1.5 and *P<0.05). The green and red colors indicate up-regulated and down-regulated transcripts, respectively. (**D**) The correlation between EMP3 and CD13 expression in GC patients with CDDP treatment was supervised by Pearson correlation analysis. (**E**) EMP3 expression was identified by Western blot assay in CDDP-resistant GC cells after CD13 was over-expressed. (**F**) CD13 expression was also determined by Western blot assay in CDDP-resistant GC cells after EMP3 was over-expressed. (**G**) Methylation-specific PCR for EMP3 in CDDP-resistant GC cells. Bands M and U represented methylated and unmethylated EMP3, respectively (**H**) The methylation status of EMP3 CpG island promoter was clarified in CDDP-resistant GC cells with indicated treatment. The results are represented as bisulfite genomic sequencing of 3 individual clones in a 4-tiered semi-quantitative grey-scale pattern (left panels): white square, represented not methylated, and methylation score was “0”; light gray, represented weakly methylated and methylation score was “1”; gray represented moderately methylated and methylation score was “2”; black represented strongly methylated and methylation score was “3”. Means±SD of methylation score (right panel) from three independent experiments were also shown. **P<0.01. (**I**) CDDP-resistant GC cells were pre-transfected with pEGFP-N1-CD13 or NC plasmids for 24 h, and then stimulated with Ubenimex (0.2mg/mL) for another 24 h. EMP3 expression were detected by Western blot assay. (**J**, **K**) Direct interaction between endogenous CD13 and EMP3 in MKN-45/DDP (**J**) and Ubenimex-treated MKN-45/DDP cells (**K**) were determined by co-IP assays using anti-CD13 or anti-EMP3. For Western blot assay, all of the data were manifested as the representatives and the relative expression with means±SD from three independent experiments. **P < 0.01 and #P>0.05.

To further illustrate the mechanism by which Ubenimex reverses chemoresistance in CDDP-resistant GC cells, we screened for differentially expressed genes (DEGs) in Ubenimex-treated MKN-45/DDP cells compared to MKN-45/DDP cells using mRNA microarray analysis supplied by Affymetrix Array Strips. Detailed information for all DEGs were supplied as the Supplementary Databases 1 and 2. Approximately 749 up-regulated and 557 downregulated mRNAs (Fold Change ≥ 1.5) in the Ubenimex-treated MKN-45/DDP cells were identified (Figure 5A–5C). As discussed previously, CD13 is a direct target for Ubenimex that reverses CDDP resistance, so we aimed to identify the downstream genes of CD13 based on the correlation between the expression of CD13 and these DEGs. EMP3, which belongs to the down-regulated clusters, gained more attention because its expression in GC patients with CDDP-based chemotherapy was positively associated with the CD13 protein level (Figure 5D). In addition, CD13 over-expression and knockdown, can up-regulated and down-regulated the EMP3 expression, respectively, but the change of CD13 expression was not detected in CDDP-resistant GC cells after EMP3 was over-expressed and silenced (Figure 5E, 5F and Supplementary Figure 5). More importantly, CD13 silence and Ubenimex treatment both induced EMP3 CpG island hypermethylation, but this effect mediated by Ubenimex was restrained after CD13 over-expression (Figure 5G and 5H). Consequently, Ubenimex remarkably suppressed EMP3 expression in CDDP-resistant GC cells, but it have no effect on EMP3 expression after CD13 is over-expressed (Figure 5I). Co-IP assay further verified that there was a direct interaction between CD13 and EMP3 protein, however, the association of CD13 and EMP3 were reduced in MKN-45/DDP cells after Ubenimex treatment (Figure 5J and 5K). Therefore, we can propose that CD13 has a one-way regulatory effect on EMP3 expression by holding its methylation back. These findings also demonstrated that EMP3 may be the candidate gene downstream of CD13 in CDDP-resistant GC cells and Ubenimex stimulation that can induce hypermethylation-mediated transcriptional silencing of EMP3.

### CD13-dependent reduction of EMP3 induced by Ubenimex attenuates the activity of the PI3K/AKT/ NF-κB pathway in CDDP-resistant GC cells

GO and KEGG analyses were then performed to achieve gene co-expression and pathway enrichment. GO analysis showed that the DEGs in Ubenimex-treated MKN-45/DDP cells were implicated in the intracellular signal transduction induced by enzyme or receptor-binding, as well as the regulation of cell death, adhesion, and movement (Supplementary Figure 6A). KEGG pathway analysis confirmed that PI3K signaling pathway is typically gene-enriched in MKN-45/DDP cells with Ubenimex treatment (Supplementary Figure 6B). EMP3 overexpression has been shown to be involved in the accumulated activation of the PI3K/AKT pathway [20]. Thus, we hypothesized that Ubenimex disturbs the PI3K/AKT pathway by targeting the CD13/EMP3 axis in CDDP-resistant GC cells.

**Figure 6:**
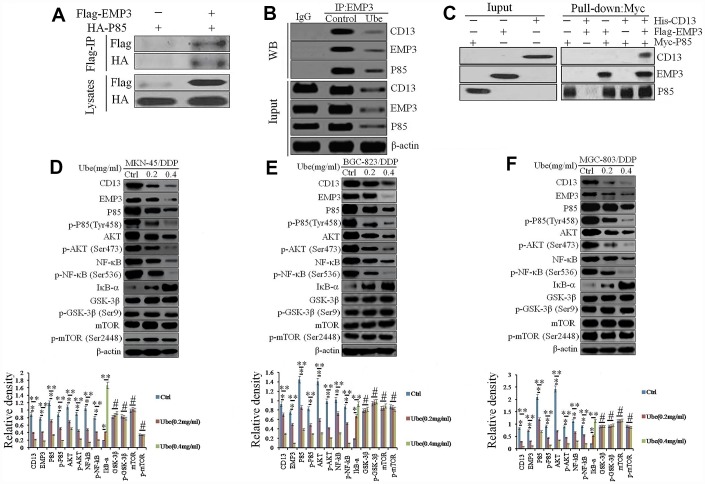
**Ubenimex inhibits CD13 expression to modulate the EMP3/PI3K/AKT/NF-κB pathway in CDDP-resistant GC cells.** (**A**, **B**) Co-IP assay using anti-Flag or anti-HA antibodies was performed in MKN-45/DDP (**A**) or Ubenimex-treated MKN-45/DDP cells (**B**). (**C**) Pull-down assay using anti-His, anti-Flag or anti-Myc antibodies was executed in HEK-293 cells that were transfected with the combination of p3×FLAG-CMV-14-EMP3, pET302 NT-His-CD13 and pCMV6-Myc-P85 plasmids. (**D**–**F**) MKN-45/DDP cells (**D**), BGC-823/DDP (**E**) and MGC-803/DDP (**F**) cells were treated with Ubenimex (0.2 or 0.4 mg/mL) for 24 h. Western blot assay was carried out to confirm the expression of the signaling molecules in the PI3K/AKT/NF-κB pathway. Data are represented as the representatives (upper panels), as well as the means±SD of relative intensities that were normalized to β-actin or corresponding total proteins (bottom panels) from three independent experiments *P<0.05, **P<0.01 and ^#^P>0.05.

As expected, P85 was detected in anti-Flag immuno-precipitates, which indicated a direct co-localization between EMP3 and PI3K in MKN-45/DDP cells, however, it was attenuated following Ubenimex administration (Figure 6A and 6B). Pull-down assay corroborated that His-CD13 can only be captured by Myc-P85 in the presence of Flag-EMP3 (Figure 6C), suggesting that the interaction between CD13 and PI3K is indirect and EMP3 dependent.

As displayed in Supplementary Figure 7, the genes enriched in the PI3K signaling pathway in Ubenimex-treated MKN-45/DDP cells included PIK3R1, AKT1, NFKBIA, RELA, etc. Consistently, Ubenimex markedly reduced the total amount of PI3K (P85) and phospho-PI3K (p-P85), as well as endogenous AKT and phospho-AKT in CDDP-resistant GC cells (Figure 6D–6F). Additionally, IκB-α expression was increased, whereas NF-κB and p-NF-κB were significantly decreased after CD13 and EMP3 expression were inhibited by Ubenimex (Figure 6D–6F). These results show that Ubenimex disrupted the PI3K/AKT/NF-κB pathway, in which the interaction between CD13 and EMP3, as well as EMP3 and PI3K are decreased.

### Ubenimex suppresses autophagy to promote CDDP-induced apoptosis in GC cells by inhibiting the activation of the CD13/EMP3/PI3K/AKT/ NF-κB pathway

**Figure 7:**
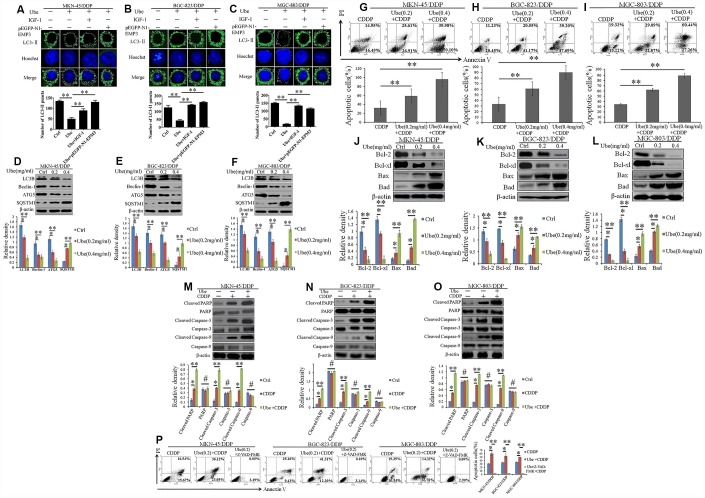
**Ubenimex inhibits autophagy and activates CDDP-induced apoptosis in GC cells via suppressing activation of the CD13/EMP3/PI3K/AKT/NF-κB pathway.** (**A**–**C**) CDDP-resistant GC cells were pre-transfected with pEGFP-N1-EMP3 for 24 h or pre-stimulated with IGF-1 (10 ng/mL) for 8 h, and then treated with Ubenimex (0.2 mg/mL) for another 24 h. LC3-II distribution in MKN-45/DDP (**A**), BGC-823/DDP (**B**) and MGC-803/DDP (**C**) cells were determined via a confocal microscopy, and were represented as the stained granules (upper panels). The number of LC3-II puncta with the means±SD (bottom panels) were also calculated (bottom panels). **P<0.01. (**D**–**F**) CDDP-resistant GC cells were treated with Ubenimex (0.2 or 0.4 mg/mL) for 24 h, expression of autophagy-related markers in MKN-45/DDP (**D**), BGC-823/DDP (**E**) and MGC-803/DDP (**F**) cells were identified via Western blot assay. (**G**–**I**) CDDP-resistant GC cells were pre-stimulated with Ubenimex, and then treated with CDDP. Apoptosis in MKN-45/DDP (**G**), BGC-823/DDP (**H**) and MGC-803/DDP cells (**I**) were evaluated using Annexin V/PI staining. (**J**–**L**) Indicated cells were treated with Ubenimex (0.2 or 0.4 mg/mL) for 24 h, and then stimulated with CDDP (20 μmol/L) for another 48h, the expression of apoptosis related proteins in MKN-45/DDP cells (**J**), BGC-823/DDP (**K**) and MGC-803/DDP cells (**L**) were detected using Western blot assay. (**M**–**O**) CDDP-resistant GC cells were pre-treated with Ubenimex (0.2 mg/mL) for 24 h, followed by the stimulation with CDDP (20 μmol/L) for another 48 h. The expression of total and cleaved PARP, Caspase-3 and Caspase-9 in MKN-45/DDP cells (**M**), BGC-823/DDP (**N**), and MGC-803/DDP cells (**O**) were examined by Western blot assay. (**P**) CDDP-resistant GC cells were pre-stimulated with Ubenimex, and they were treated with Z-VAD-FMK (50μM) for another 2 h before CDDP administration. Cell apoptosis were evaluated using Annexin V/PI staining. For cell apoptosis analysis, data are demonstrated as the representatives (upper or left panels), as well as the proportions of apoptotic cells with the means±SD (bottom or right panels) from three independent experiments, *P<0.05 and **P<0.01. For Western blot assay, data are displayed as the representatives (upper panels) and the means±SD (bottom panels). *P<0.05, **P<0.01and #P>0.05.

We next investigated whether Ubenimex reverses CDDP resistance in GC cells by interfering autophagy. LC3II addiction on autophagic membrane has been widely considered to be a hallmark of autophagosome formation [21]. Herein, our data demonstrated that the number of LC3-II protein significantly decreased in CDDP-resistant GC cells after being incubated with Ubenimex (Figure 7A–7C). However, pEGFP-N1-EMP3 plasmid and a constitutive activator of PI3K/AKT pathway IGF-1, both restored the autophagosome formation, represented as increased LC3-II staining (Figure 7A–7C). Furthermore, the down-regulation of LC3B, Beclin-1, and ATG5, and the up-regulation of SQSTM1 were detected in CDDP-resistant GC cells after Ubenimex treatment (Figure 7D–7F).

A recent study has shown that autophagy helps tumor cells evade apoptosis and thus induce chemoresistance in tumor cells [22]. Herein, AnnexinV FITC/PI staining indicated that Ubenimex significantly increased CDDP-induced apoptosis in CDDP-resistant GC cells (Figure 7G–7I). As generally acknowledged, dys-regulated expression of B-cell lymphoma 2 (Bcl-2) family followed by the inactivation of Caspase-3 or Caspase-9 are the main cause of apoptosis resistance in GC cells [23, 24]. Consistent with this, Ubenimex decreased the expression of Bcl-2 and Bcl-xl, but it up-regulated the expression of Bax and Bad (Figure 7J–7L). As shown in Figure 7M–7O, Ubenimex also distinctly potentiated the expression of cleaved forms of PARP, Caspase-3, and Caspase-9. Moreover, the promotion of CDDP-induced apoptosis mediated by Ubenimex could be blocked by the pan-caspase-inhibitor Z-VAD-FMK (Figure 7P). Taken together, these findings show that Ubenimex encourage CDDP-induced apoptosis, which is Caspase-3/9 dependent, in part by autophagy inhibition, in which suppression of the activity of the CD13/EMP3/PI3K/AKT/NF-κB pathway is necessary.

### Ubenimex restrained EMT process to reduce migratory and invasive abilities of CDDP-resistant GC cells by alleviating the activation of the CD13/EMP3 /PI3K/AKT/NF-κB pathway

**Figure 8:**
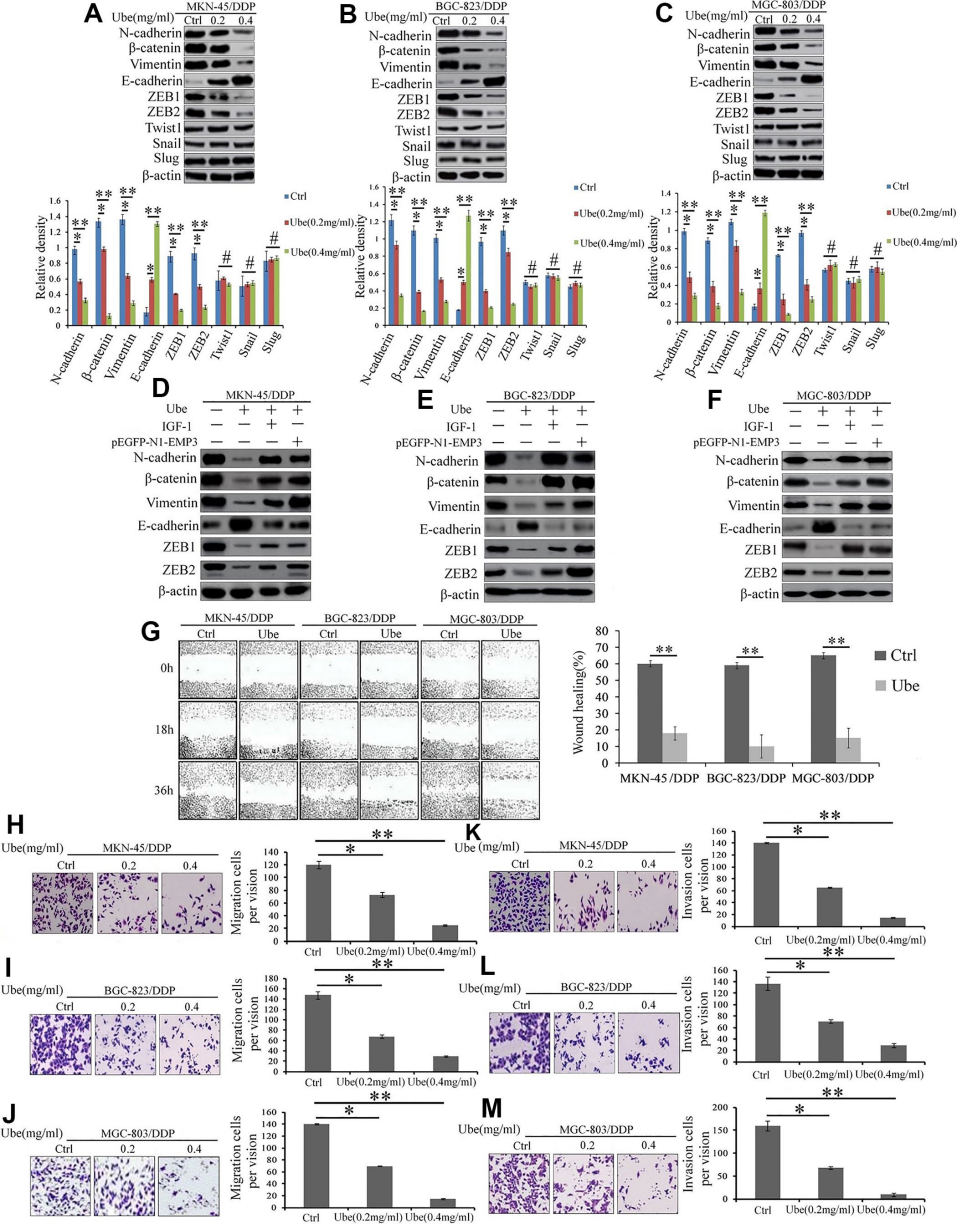
**Ubenimex suppresses the EMT, migration, and invasion of CDDP- resistant GC cells by attenuating the activation of the CD13/EMP3/PI3K/AKT/NF-κB pathway.** (**A**–**C**) Western blot assay was employed to explore the expression of EMT markers in MKN-45/DDP (**A**), BGC-823/DDP (**B**) and MGC-803/DDP cells (**C**), which were stimulated with Ubenimex (0.2 or 0.4 mg/mL) for 24 h. Data are expressed as the representatives (upper panels), and relative expression with means ±SD (bottom panels). **P<0.01,*P<0.05 and #P>0.05. (**D**–**F**) Indicated cells were pre-treated with pEGFP-N1-EMP3 plasmid for 24 h or IGF-1 (10ng/mL)or 8h, followed by treatment with Ubenimex (0.4mg/mL) for another 24 h, indicated EMT markers in MKN-45/DDP cells (**D**), BGC-823/DDP (**E**) and MGC-803/DDP (**F**) cells were detected by Western blot assay. (**G**) Wound healing assays were carried out to determine the migration abilities of CDDP-resistant GC cells which were treated with Ubenimex. Cell morphology of gap with different widths at 0, 18, and 36 h were obtained (left panels), and data are summarized as the means ± SD of “healing ratio”(right panel) **P<0.01. (**H**–**M**) Transwell assays was performed to identify the changes of the migration (**H**–**J**) and invasive (**K**–**M**) abilities in CDDP-resistant GC cells which were treated with Ubenimex (0.2 or 0.4 mg/mL) for 24h. Results were shown as the representatives (left panels) and the number of migrated and invasive cells with the means±SD (right panels) from three experiments. *P<0.05 and **P<0.01.

To clarify whether Ubenimex abolishes CDDP resistance in GC cells by blocking EMT, the expression of EMT indicators were first examined. Figure 8A shows that there is a considerable reduction in N-cadherin, β-catenin and Vimentin expression, but a significant increase in E-cadherin expression in Ubenimex-treated CDDP-resistant GC cells compared to the control cells. Furthermore, EMT-related transcription factors ZEB1 and ZEB2, but not Twist1, Snail, and Slug were significantly down-regulated in CDDP-resistant GC cells following Ubenimex stimulation (Figure 8A–8C). EMP3 overexpression and IGF-1 administration both offset the regulatory effect of Ubenimex on the expression of those EMT-related markers (Figure 8D–8F). EMT is recognized as a vital step during the early stage of metastasis and tumor cells with EMT acquires a high potential for migration and invasion [25]. Here, wound healing assay revealed that CDDP-resistant GC cells treated with Ubenimex showed lower motility than the untreated cells (Figure 8G). Similarly, transwell assays indicated that the mobility and invasiveness were effectively suppressed in CDDP-resistant GC cells after Ubenimex treatment (Figure 8H–8J and Figure 8K–8M). Collectively, these results indicate that Ubenimex inhibits EMT development, which is monitored by the CD13/EMP3/ PI3K/AKT/NF-κB axis, followed by decreased metastasis of CDDP-resistant GC cells.

## DISCUSSION

To the best of my knowledge, our results serve as the first demonstration that CD13 upregulation contributes to chemoresistance in GC cells, probably causing chemotherapy failure and poor prognosis in GC patients with CDDP treatment. Interestingly, our findings showed that Ubenimex reverses CDDP resistance, and it also had a synergetic anti-tumor effect with CDDP *in vitro and in vivo* by directly down-regulating CD13 expression. Although EMP3 was considered as a positive force on the activation of the PI3K/AKT pathway in tumor cells [20.26], investigations on EMP3 and CDDP-resistant GC, as well as the key mechanisms of which EMP3 enhanced the activity of the PI3K/AKT pathway in GC cells are limited. The present study has identified that the aberrant expression of EMP3 in GC cells can be partly attributed to the cripple of hypermethylation to its silencing mechanisms, and CD13 may be an evil initiator of this process. Also, this is the first study to elucidate that CD13 functions upstream of EMP3 to induce its expression, and EMP3 up-regulation facilitates optimal phosphorylation of P85 to activate the PI3K/AKT/NF-κB pathway. Based on these conclusions, we confirm that Ubenimex can reverse CDDP resistance and enhance CDDP-sensitivity by inhibiting the activation of the CD13/EMP3/PI3K/AKT/NF-κB pathway in GC cells.

Enhanced drug efflux [27] and reduced cell death [28] caused by overstimulation of the PI3K/AKT pathway are well-known forces that trigger CDDP resistance in GC cells. Herein, our results reveal that Ubenimex reverses CDDP resistance in GC cells, partly by suppressing autophagy and EMT development, in which inactivation of the CD13/EMP3/PI3K/AKT/NF-κB pathway is indispensable. Thus, we present a new evidence that over-activation of the PI3K/AKT/NF-κB pathway was promoted by CD13/EMP3 axis in GC cells, thus conferring CDDP resistance, probably by facilitating autophagy and EMT development.

In the canonical starvation-induced pathway of autophagosome formation, the initiation step is negatively regulated by the PI3K/AKT mTOR pathway [29], vesicle nucleation is processed by Beclin-1, which is required for the formation of the ClassIIIPI3K complex [30], and vesicle elongation step is monitored by LC3, ATG5, and SQSTM1 [31]. Recent reports have suggested that NF-κB, a well-known transcription factor, can stimulate autophagy by upregulating the expression of Beclin-1, LC3, ATGs, and SQSTM1 [32–33]. Thus, it is plausible that Ubenimex inhibits autophagy in GC cells by suppressing the activity of PI3K and AKT, resulting in the impediment of IkB-α degradation and NF-κB phosphorylation, and the down-regulation of LC3B, Beclin-1, and ATG5, but increased expression of SQSTM1. Therefore, our findings disclosure a possibility that besides mTOR, NF-κB is also another regulatory element downstream of PI3K/AKT that induces autophagy in GC cells.

Autophagy is characterized as a double-edged sword that plays roles in pro-death and pro-survival, processes that are associated with the interaction of apoptosis in GC cells [34]. Usually, autophagy lies on the upstream of apoptosis and is required for apoptotic cell death in chemo-sensitive GC cells [35]. However, other machinery talk between autophagy and apoptosis in chemo-resistant GC cells has been well established, in which autophagy serves as a oncogenetic factor that favors the escape of cell apoptosis [35, 36]. As demonstrated in our study, CDDP-resistant GC cells also had a poor response to CDDP-induced apoptosis, but exhibited apparent autophagic property (Figure 7). Some autophagy inhibitors, such as 3-MA and chloroquine, can both sensitize the cytotoxicity of CDDP and apparently induce the apoptosis of GC cells [37, 38]. As the protagonist of our study, Ubenimex also can be an reliable autophagy inhibitor to promote CDDP-induced apoptosis.

EMT-mediated chemoresistance also represents one mechanism underlying CDDP resistance in GC. A oxaliplatin-resistant GC cell line SCG-7901/OXA, MDR GC cell line BGC-823/X, and Lapatinib-resistant HER 2-positive MGC-803 cells all showed a EMT phenotype with more powerful capability of migration and invasion [39]. Inhibition of N-cadherin, β-catenin, Snail, Twist, Zeb1, and Zeb2 expression increased GC cells sensitivity to chemotherapeutic drugs [40]. ZEB1 and ZEB2 expression were also higher in GC patients treated with a combination of capecitabine and oxaliplatin, relatively to that with no chemotherapy application [41]. Consistently, our results also showed that Ubenimex alleviated EMT in CDDP-resistant GC cells through rearranging the expression of EMT-related molecules to suppress tumor invasion and migration.

AKT has been proved to be an important initiator to induce EMT by increasing ZEB1 and ZEB2 expression [42], the subsequent inhibition of EMT can be reversed by IGF-I, suggesting that the activation of the PI3K/ AKT//ZEB2 pathway dominates the EMT development in GC cells [43]. Via activating the AKT/Ikk-α/NF-κB pathway, Zipper-interacting protein kinase (ZIPK) increased Zeb1, Zeb2 and Snail expression, but decreased the protein level of E-cadherin to accelerate EMT progress in GC patients [44]. In view of the above, it is reasonable and innovative that Ubenimex impeded EMT process by depressing the activity of the CD13/EMP3/PI3K/AKT/NF-κB pathway in GC cells.

In conclusion, our findings indicate that poor responses to CDDP-based chemotherapy might be resolved by autophagy and EMT inhibition at least in part by interfering with the CD13/EMP3/PI3K/AKT/NF-κB axis. Our data also provided a comprehensive insights into the combination of Ubenimex and CDDP, which may represent a novel therapeutic approach for GC patients.

## MATERIALS AND METHODS

### Chemicals and antibodies

Ubenimex (Ube) was obtained from Shenzhen Main Luck Pharmaceutical, Inc. (Shenzhen, China). CDDP and recombinant human insulin-like growth factor-1 (IGF-1) were purchased from R&D Systems, Inc. (Minneapolis, MN, USA). The details on indicated antibodies are described in Supplementary Materials and Methods.

### Cell lines and cell culture

Human GC cell lines (MKN-45, BGC-823, and MGC-803) were obtained from Cell Bank of the Chinese Academy of Sciences (Shanghai, China), HEK-293 and human gastric epithelia cell line GES1 were purchased from the American Type Culture Collection (Manassas, VA, USA). These cells were maintained in RPMI 1640 medium (Gibco, Grand Island, NY, USA), which was supplemented with 15% fetal calfserum (Gibco, NY, USA). The cells were cultured at 37°C in a humidified atmosphere with 5% CO_2_.

### Subjects and tissue samples

Tissue samples were collected from 195 GC patients, which consisted of 91 males and 104 females, of which their ages ranged from 45 to 67 years. All of the patients had received CDDP-based chemotherapy at the Affiliated Hospital of Qingdao University between February 2006 and December 2011, and they were divided into two groups according to the response to CDDP-based chemotherapy. GC patients who exhibited complete or partial remission were classified as the CDDP-sensitive group. In particular, complete remission was defined as the disappearance of all target lesions or the deficiency of new focus for at least four weeks; partial remission refers to a decrease of lesion size by more than 30% for at least four weeks. Alternatively, the CDDP-resistant group was composed of those patients with progressive disease. Patients with progressive disease exhibited an increase in tumor size by at least 20% or novel intragastric lesions. Normal gastric mucosa was acquired from volunteers with non-neoplastic disease from the organ donor center of the Affiliated Hospital of Qingdao University and was used as the controls. All of the tissue specimens were preserved in liquid nitrogen for Western blot assay, or were paraffin-embedded for immunohistochemical staining. Approval from the Ethics Committee of Qingdao University was obtained for this study, and informed written consent was obtained from each patient.

### Establishment of CDDP-resistant GC cells

Parental MKN-45, BGC-823, and MGC- 803 at the logarithmic growth phase were seeded in the culture medium containing CDDP at an initial concentration of 0.05 μg/mL. Every four days later, surviving cells were re-collected and cultured with fresh medium containing double concentration of CDDP, which can be supplied at the highest concentration of 1 μg/mL. About six months later, CDDP-resistant GC cells, named as MKN-45/DDP, MGC-803 /DDP, and BGC-823/DDP cells, were successfully generated.

### Cytotoxicity assay and determination of drug resistance

The indicated cells were administrated with Ubenimex (0.2 mg/mL) or/and pEGFP-N1-CD13 transfection for 24 h, and then stimulated with CDDP at gradient concentration for another 48 h. To evaluate drug resistance, the CDDP-resistant GC cells and their parental cells were pre-treated with indicated plasmids or Ubenimex (0.2 mg/mL) for 24 h, followed by the stimulation with CDDP (0, 2, 4, 8, and 16 μg/mL) for another 48 h. Finally, the cells were incubated with CCK-8 reagent for 2h, and cell absorbance at a wavelength of 450 nm were read by a microplate reader (Bio-Rad, USA). The inhibition rate, 50% inhibitory concentration (IC50) values, and resistance indices (RIs) were calculated according to the formulas as described in our previous report [45].

### Plasmid construction and lentiviral packaging

Construction of pTZU-CD13-shRNA, pEGFP-N1-CD13, pTZU-EMP3-shRNA and pEGFP-N1-EMP3 plasmids was carried out according to previously described methods [46]. In addition, human EMP3 cDNA was cloned into a p3× FLAG-CMV-14 vector (Sigma-Aldrich) in line with the methods as described elsewhere [47]. PCMV-HA-P85, pET302 NT-His-CD13, and pCMV6-Myc-P85 were purchased from Sangon Biotech Co., Ltd. (Shanghai, China). All of the plasmids were delivered into cells using Lipofectamine™ 3000 Transfection Reagent (Invitrogen) according to the manufacturer’s instructions. PEGFP-N1-CD13 plasmid was further packed with lentiviral packaging systems. Briefly, pEGFP-N1-CD13 plasmid was linearized into pGCSIL-GFP lentiviral vectors, which were subsequently transfected into HEK-293 cells. After incubating for 72 h, the culture supernatants were collected and filtered, in which viruses were captured via centrifugation at 4°C for 1 h. Harvested viruses containing the recombinant plasmid LV-pEGFP-N1-CD13 were stored in liquid nitrogen.

### Xenograft models and treatment

BALB/c male nude mice (4-6 weeks old) were purchased from Vital River Laboratory Animal Technology Co., Ltd. (Beijing, China), and they were kept under specific pathogen-free (SPF) conditions. Approximately 1×10^7^ MKN-45/DDP cells at the logarithmic growth phase were subcutaneously inoculated into right flanks of each mouse. After two weeks, the tumor-bearing mice were randomly divided into four groups and treated as follows: (1) the Control group, which was treated with PBS; (2) Ubenimex-treatment group which was intratumorally administrated with Ubenimex (20mg/kg) every three days for four weeks;(3) the CDDP-treatment group, which was given CDDP at 4 mg/kg every three days via intratumoral injection for four weeks; and (4) the Combination group, which was managed with the alternative treatment of CDDP and Ubenimex, i.e., the mice were pre-treated with Ubenimex (20 mg/kg) by intratumoral injection, three days later, CDDP (4 mg/kg) administration was followed. Such a cycle was maintained for four weeks. To over-express CD13 in CDDP-treatment and combination group, LV-pEGFP-N1-CD13 plasmid (MOI =60) was dissolved in Lentivirus Enhancement Reagent Envirus TM-LV (Engreen Biosystem Co, Ltd.), and was administered intravenously 12 hours before each treatment. After another 2 weeks, the mice were sacrificed, and tumor volume was calculated by length×width^2^/2 and tumor weight was measured. Finally, tumor specimens were kept at -80°C for Western blot assay. All experimental studies were approved by the Qingdao University Animal Care and Use Committee.

### MRNA microarray analysis

Total RNA of MKN-45/DDP cells with or without Ubenimex treatment was isolated using E.Z.N.A. Total RNA Kit I (Omega Bio-Tek), according to the manufacturer’s instructions. MRNA expression profiling was performed by human Clariom™ S Assay using the GeneChip® 3′ IVT Pico Kit (Affymetrix). Detailed information is supplied in the .

### Western blot assay

Western blot assay was performed as previously described [46]. Detailed information is provided in the Supplementary Materials and Methods.

### Cell apoptosis analysis by flow cytometry

CDDP-resistant GC cells were pre-treated with Ubenimex (0.2 or 0.4 mg/mL) for 24 h and they were stimulated with CDDP (20 μmol/L) for another 48 h. Alternatively, CDDP-resistant GC cells were pretreated with pan- caspase-inhibitor-Z-VAD-FMK (50 μM) (Promega, USA) for 2 h before CDDP administration. Apoptosis analysis was performed as previously described [46].

### Wound healing assay

CDDP-resistant GC cells were seeded in six-well culture plates and treated with Ubenimex (0.2 mg/mL) for 24 h. When the cells had reached 100% confluency, the culture medium was discarded. Then, the cell monolayer was scratched to introduce a gap, to which fresh serum-free medium was added. The gaps were photographed at 0, 18, and 36 h since scratching, and the migration ability was represented as the percentage closure of the gap that was measured usi ng Celigo® Image Cytometer (Nexcelom, USA).

### Transwell chamber assays

The transwell chamber assay was conducted using Transwell insert (8-mm pore size, Corning, USA), for cell invasion assays, the Transwell insert was covered with Matrigel (BD Bioscience). Briefly, 1×10^5^ cells were suspended in the top chamber with 200 μL of serum-free medium, whereas 800 μL of the culture medium containing 15% FBS were added into the bottom chamber. After incubating for 24 h, the cells that had migrated or invaded the underside of the membranes were fixed in 4% polyformaldehyde, stained with 0.5% crystal violet, and counted using a light microscope.

### Co-immunoprecipitation (Co-IP) and pull-down assay

Co-immunoprecipitation and pull-down assay were conducted according to the methods described in the Supplementary Materials and Methods.

### Immunohistochemistry (IHC) analysis

The slides from tissue specimens were blocked in the 0.3% BSA for 1 h, and then incubated with the primary antibody against CD13 (1:500 dilution) at 4°C overnight. After staining with goat anti-rabbit IgG H&L (HRP) (1:100 dilution) at 37°C for1 h, the sections were treated with DAB solution at 37°C for 15 min, followed by observation under a light microscope. The brown or brownish-yellow particles were considered as the tumor cells that were positive-staining. Image-Pro Plus V6.0 software was used for image analysis and staining scores were obtained based on the percentage of colored particles that formed a thin and brown-yellow layer around the whole cell membrane. For each coverslip, the proportion of CD13-positive cells ranged from 0% to 100%, and intensity scores varied from 0 to 3, with 0 indicating no staining or staining with<25%; 1, weak staining with 25-50% positive (+); 2, moderate staining with 50-75% positive (++); and 3, strong staining with 75-100% positive (+++).

### Autophagy evaluation by LC3-II puncta formation

The CDDP-resistant GC cells were fixed with 0.4% paraformaldehyde for 30 min at room temperature, and then treated with 0.1% Triton X-100 and blocked with 5% (w/v) non-fat dry milk for another 30 min. After washing with PBS, the cells were probed with LC3-II unconjugated antibody (1:500 dilution, ab48394, Abcam) overnight at 4°C. Then, the treated cells were washed thoroughly and incubated with Alexa Fluor® 488-conjugated antibodies (1: 500, ab150073, Abcam) for 40 min at 37°C, followed by the staining with Hoechst 33342 for another 10 min. Fluorescent protein complexes were observed under a laser scanning confocal microscope (OLS4500, OLYMPUS), and the number of membrane-bound LC3 II was counted and summarized by Image Pro Plus V6.0 software.

### Methylation-specific PCR (MS-PCR) analysis

Total RNA of indicated cells was extracted using the TRizol regent (Invitrogen) and cDNAs were synthesized using high capacity cDNA synthesis kit (Invitrogen, Grand Island, NY, USA). Semi-quantitative RT-PCR was performed as previously described [46], using primers specific for the methylated or modified unmethylate DNA. CpGenome Universal Methylated DNA (Chemicon International Inc., Temecula, CA, USA) and normal lymphocyte (NL) DNA were used as methylated and unmethylated controls, respectively.

Primer sequences of EMP3 for the unmethylated reaction: Forward: 5′-GAAGAGATGTAGAAGGAGA GTGAGT-3′; Reverse:5′-CTTATCCCTCACTCAAAC CTCCATA-3′. Primer sequences of EMP3 for the methylated reaction: Forward:5′-GACGTAGAAGGAG AGCGAGC-3′; Reverse:5′-CCTCGCTCGAACCTCCG TA-3′. Alternatively, cDNA was treated with sodium bisulfite. Methylation status was analyzed by bisulfite genomic sequencing of the EMP3 CpG island. 12 CpG-sites were evaluated for methylation within a 300-basepair amplicon encompassing the EMP3 transcription start site. The primer sequences were as follows: Forward:5′-TAGTATATATTGAGAGGAGGAGAG-3′; Reverse:5′-CTTCCCAAACTACTACATTCCCA-3′. PCR products were recovered and sequenced using the BigDye Cycle Sequencing kit v1.1 (Applied Biosystems). Methylation scores were calculated using GeneMapper v4.0 software for fragment analysis (Applied Biosystems), and were determined according to the peak height ratio between peaks for the methylated and unmethylated allele: score 0:no methylation; score 1:weak methylation;score 2:moderate methylation; score 3:strong methylation. The methylation status were subdivided into two groups: (1) no EMP3 hypermethylation (methylation score 1, 2, and 3 in less than 50% of the 12 CpG-sites) (2) EMP3 hypermethylation (methylation score 1, 2 or 3 in ≥50% of the 12 CpG sites).

### Quantitative Real-time PCR

Quantitative Real-time PCR were performed according to the methods described in the Supplementary Materials and Methods.

### Statistical analysis

Comparisons between the two groups were performed using the student t test, χ^2^ test and ANOVA. Pearson correlation analysis was used to confirm the relationship between EMP3 and CD13 expression. The associations between CD13 expression and OS duration were assessed using Kaplan-Meier curves and the log-rank test. Statistical analysis of Microarray data was summarized using the t test, ANOVA and Fisher’s exact test. All of the calculations were performed using SPSS 17.0 software (SPSS Inc., Chicago, IL, USA). The level of significance was set at *P < 0.05 and **P< 0.01.

## Supplementary Materials

Supplementary Methods

Supplementary Figures

Supplementary Database 1

Supplementary Database 2
