# Supplementary material for: Ubenimex induces autophagy inhibition and EMT suppression to overcome cisplatin resistance in GC cells by perturbing the CD13/EMP3/PI3K/AKT/NF-κB axis
Source: Aging (Albany NY). 2019 Dec 31;12(1):80–105. doi: 10.18632/aging.102598 (PMC6977684; doi:10.18632/aging.102598)
Supplement: Supplementary Methods [file aging-12-102598-s001..pdf]

SUPPLEMENTARY METHODS

Antibodies

The following antibodies were used in our study: Rabbit anti-human CD13 (#SC-166270), anti-human Bcl-xl (#SC-8392), anti-human Bcl-2 (#SC-130307), anti-human Bax (#SC-6236), anti-human Bad (#SC-8044), anti-human Caspase-3 (#SC-271028), anti-human Caspase-9 (#SC-17784), anti-human ZEB2 (#SC-271984), anti-human Twist1 (#SC-81417) mAbs from Santa Cruz Biotechnology. Rabbit anti-human β-actin (#4970), anti-human PI3K (p85α,#13666), anti-human PARP (#9542), anti-human Cleaved PARP (#5625), anti-human AKT (#4685), anti-human mTOR (#2983), anti-human GSK-3β (#12456), anti-human NF-κB p65 (#8242), anti-human IκB-α (#4812), anti-human Phospho-Akt (Ser473) (#9018), anti-human phospho-GSK-3β (#5558), anti-human Phospho-NF-κB p65 (Ser536) (#3033), anti-human Phospho-mTOR (Ser2448)(#5536), anti-human Beclin-1 (#4122), anti-human ATG5 (#12994), anti-human SQSTM1 (#88588), anti-human N-cadherin (#13116), anti-human β-catenin (#8480), anti-human Vimentin (#5741), anti-human E-cadherin (#14472), anti-human ZEB1 (#3396), anti-human Slug (#9585), anti-human LC3B (#3868) mAbs from Cell Signalling Technology. Rabbit anti-human Snail (AF3639) from R&D Systems, Inc. Rabbit anti-human Phospho-PI3K p85 (Tyr458) (PL0304731) from PLLABS (British Columbia).

Rabbit anti-human Cleaved Caspase-9 (ab2324), anti-human Cleaved Caspase-3 (ab2302), anti-human EMP3 (ab73151) mAbs, Rabbit polyclonal to Myc tag antibody (ab9106) from Abcam. Anti-Flag Tag human Monoclonal Antibody (A02010), Anti-HA Tag human Monoclonal Antibody (A02040), Anti-His Tag human Monoclonal Antibody (A02050) from Abbkine.

mRNA microarray analysis

poly-A RNA controls, Total RNA/Poly-A RNA Control Mixture, Second-Strand Master Mix, IVT Master Mix were prepared in advance. RNA samples were employed with primers containing a T7 promoter and performed by reverse transcription reaction to synthesize first-strand cDNA, followed by Second-strand cDNA and cRNA synthesis. The cRNA is then purified and converted to biotinylated double-stranded cDNA (ds-cDNA) hybridization targets for unbiased coverage of the transcriptome using GeneAtlas® Hybridization Station (Affymetrix). Arrays were stained using Affymetrix GeneChip Fluidics Station 450 systems and scanned using GeneChip® Scanner 3000 7G (Affymetrix). Differentially expressed mRNAs were defined as having a Fold change ≥2 and a *p value<0.05. BioCarta or KEGG (Kyoto Encyclopedia of Genes and Genomes) pathway and Gene Ontology (GO) analysis were complemented by the Affy expression console software based on GSEA database.

Western blot assay

Tumor tissue was cut into pieces and executed to discontinuous Percoll Gradient Centrifugation. Briefly, tissue debris were digested with the mixture containing type I hyaluronidase (0.05g/L), type VI hyaluronidase (0.1g/L) and Dnase (0.2g/L) at 37°C for 4 h. The digestive suspension was filtered with centrifugation at 1200 rpm for 10 min, and the cell precipitation was resuspended in 40% Percoll working fluid (2ml) and carefully added to70% Percoll working fluid (2ml). Then, the mixed liquids with Percoll were prepared for centrifugation again. Particularly, the centrifugal speed is set to 600 g, the centrifugal time is set to 20 min, and the rising and falling rate is set to 5 g and 1 g, respectively. Finally, the white cloud-like cell layer is filled with lymphocytes lymphocyte, while tumor cells are lacated in the bottom layer. These tumor cells were collected and suspend in the desired buffer.

Total proteins of indicated cells were extracted using Minute™ Protein Extraction Kits (Invent Biotechnologies) and quantified using BCA Protein Assays Kits (Thermo Scientific). Protein samples with 50μg/lane were loaded and separated by 12% SDS-PAGE gels and transferred to the nitrocellulose membranes (Bio-Rad, Hercules, CA). The membranes was briefly washed and blocked with 5% (w/v) non-fat dry milk or 1 h. The blocked membranes were then incubated with primary antibodies overnight at 4°C and were washed with TBST carefully. Finally, the membrane was incubated with Goat Anti-Rabbit IgG H&L (HRP) (1:100 dilution, ab6721, Abcam) for 1h at room temperature. Immuno-reactive proteins were visualized using the ChemiDoc™ XRS+ System (Bio-Rad)with the assistance of SuperSignal West Pico Chemiluminescent Substrate (Thermo Scientific) [46]. Relative intensities of indicated proteins towards β-actin or corresponding 'total' proteins were evaluated.

Co-immunoprecipitation (Co-IP) and pull-down assay

MKN-45/DDP cells were collected and lysed on ice. Cell supernatant containing total proteins was incubated with G-Sepharose at room temperature for 2 h. The mixture was centrifuged to precipitate the agarose beads. All the Beads were washed by AminoLinkPlus Resin and Pierce Control Agarose Resin (Thermo Scientific)

over night at 4°C. Likewise, MKN-45/DDP cells with Flag-EMP3 and HA-P85 co-expression were lysed in NP40-based buffer (Sigma-Aldrich). Cell supernatant were cultured with anti-FLAG beads or anti-HA beads (Sigma-Aldrich) for another 3 h. Western blot assay was used to determine interaction between CD13 and EMP3, as well as EMP3and P85 via indicted antibodies.

For pull-down assay, HEK-293 cells were co-expressed with Flag-EMP3, His-CD13 and Myc-P85 and lysed in 1% Triton X-100 buffer. Cell supernatant were treated with indicated antibodies for 1h at 4°C, followed by incubation with protein G-Sepharose beads for another 3 h. All these beads were washed with lysis buffer, and were boiled in conditions buffer at 100°C for 5 min, followed by Western blot assay.

Real-time PCR analysis

RNA was isolated from cells using Trizol method. cDNAs were synthesized using high capacity cDNA synthesis kit (Invitrogen, Grand Island, NY, USA). The mRNA levels of CD13 were examined using iCycler IQ system (Bio-Rad) with the assistance of SYBR Green IqPCR Master Mix (MedChemExpress, USA), and were normalized towards GAPDH, The primer sequences of CD13 and GAPDH genes were demonstrated as follows: CD13 (Forward:5′-GGGGCACTAACCAGAAGTGG-3′, Reverse:5′-GGG AGACCCCACTAGGATGT -3′;) GAPDH (Forward:5′-GCTGGTGCTGAGTATTGCGT-3′, Reverse:5′-TGGG AGGTGCTGTTGAAGTC-3′).
