# Supplementary material for: Ubenimex induces autophagy inhibition and EMT suppression to overcome cisplatin resistance in GC cells by perturbing the CD13/EMP3/PI3K/AKT/NF-κB axis
Source: Aging (Albany NY). 2019 Dec 31;12(1):80–105. doi: 10.18632/aging.102598 (PMC6977684; doi:10.18632/aging.102598)
Supplement: Supplementary Figures [file aging-12-102598-s003..pdf]

SUPPLEMENTARY FIGURES

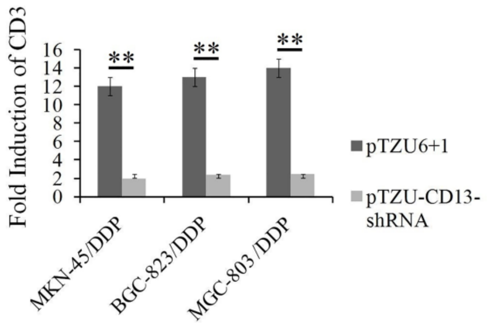

Supplementary Figure 1. Quantitative study of CD13 mRNA expression in CDDP-resistant GC cells after CD13 konckdown. CDDP-resistant GC cells were pre-stimulated with pTZU6+1 or pTZU-CD13-shRNA plasmids, Real-time PCR assay was performed to check the CD13 expression. The results are demonstrated as the representative of three independent experiments. **P<0.01.

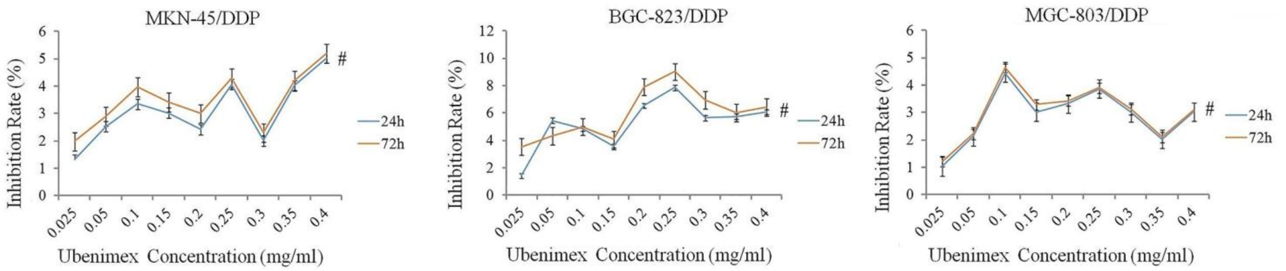

Supplementary Figure 2. Ubenimex has no direct cytotoxicity on CDDP-resistant GC cells. CDDP-resistant GC cells were treated with increasing concentrations of Ubenimex for 24h and 72h. Inhibition rate on cell growth was calculated by CCK-8 method. #P>0.05.

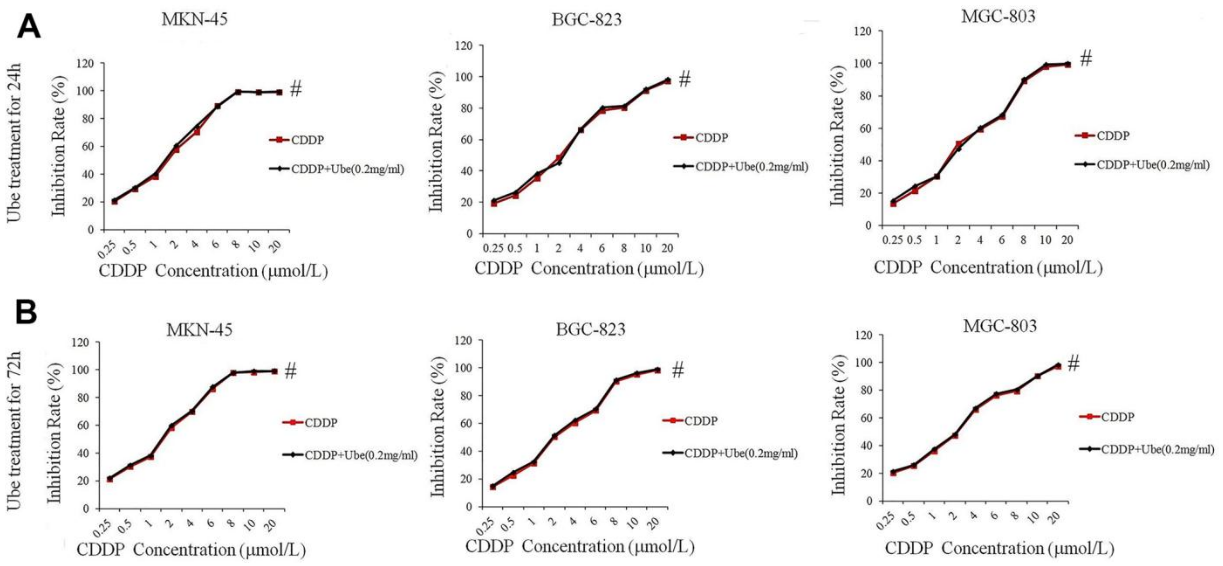

Supplementary Figure 3. Ubenimex has no effect on cytotoxicity of CDDP towards parental GC cells. GC cells were treated with or without Ubenimex (0.2mg/mL) for 24h and 72h, followed by incubation with CDDP at increasing concentrations (0.25, 0.5, 1, 2, 4, 6, 8, 10, and 20 μmol/L) for 48h. Inhibition rate on cell growth was measured with CCK-8 method. #P>0.05.

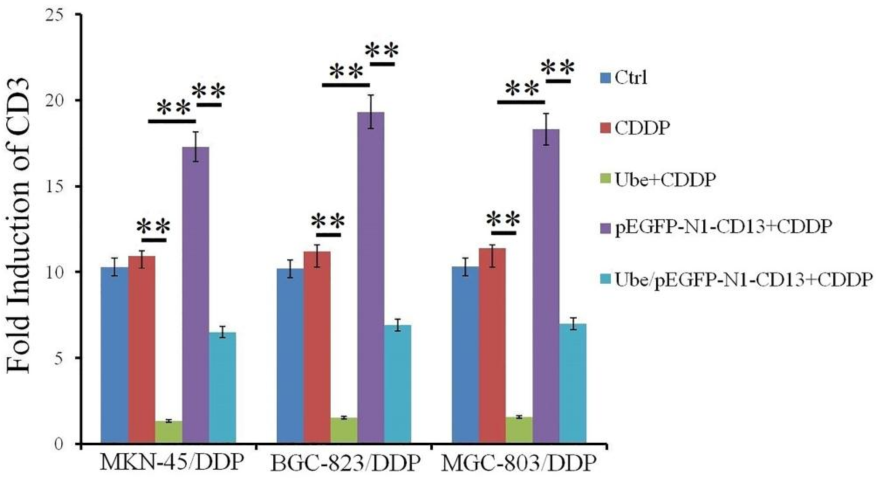

Supplementary Figure 4. Quantitative study of CD13 mRNA expression in CDDP-resistant GC cells after Ubenimex treatment. CDDP-resistant GC cells were pre-stimulated with indicated treatment as described in the Figure 3, and was prepared for Real-time PCR assay. The results are demonstrated as the representative of three independent experiments. **P<0.01.

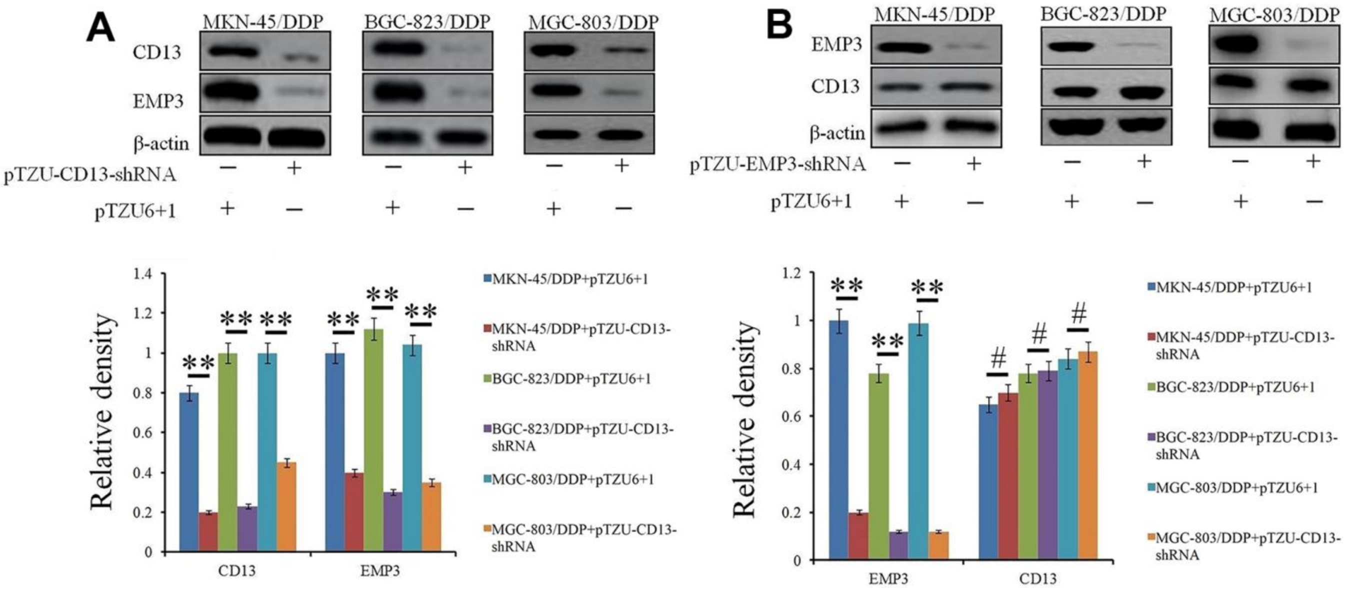

Supplementary Figure 5. CD13 has a one-way regulatory effect on EMP3 expression. (**A**) EMP3 expression was identified by Western blot assay in CDDP-resistant GC cells after CD13 was knockdown. (**B**) CD13 expression was determined by Western blot assay in CDDP-resistant GC cells after EMP3 was silenced. **P<0.01 and #P>0.05.

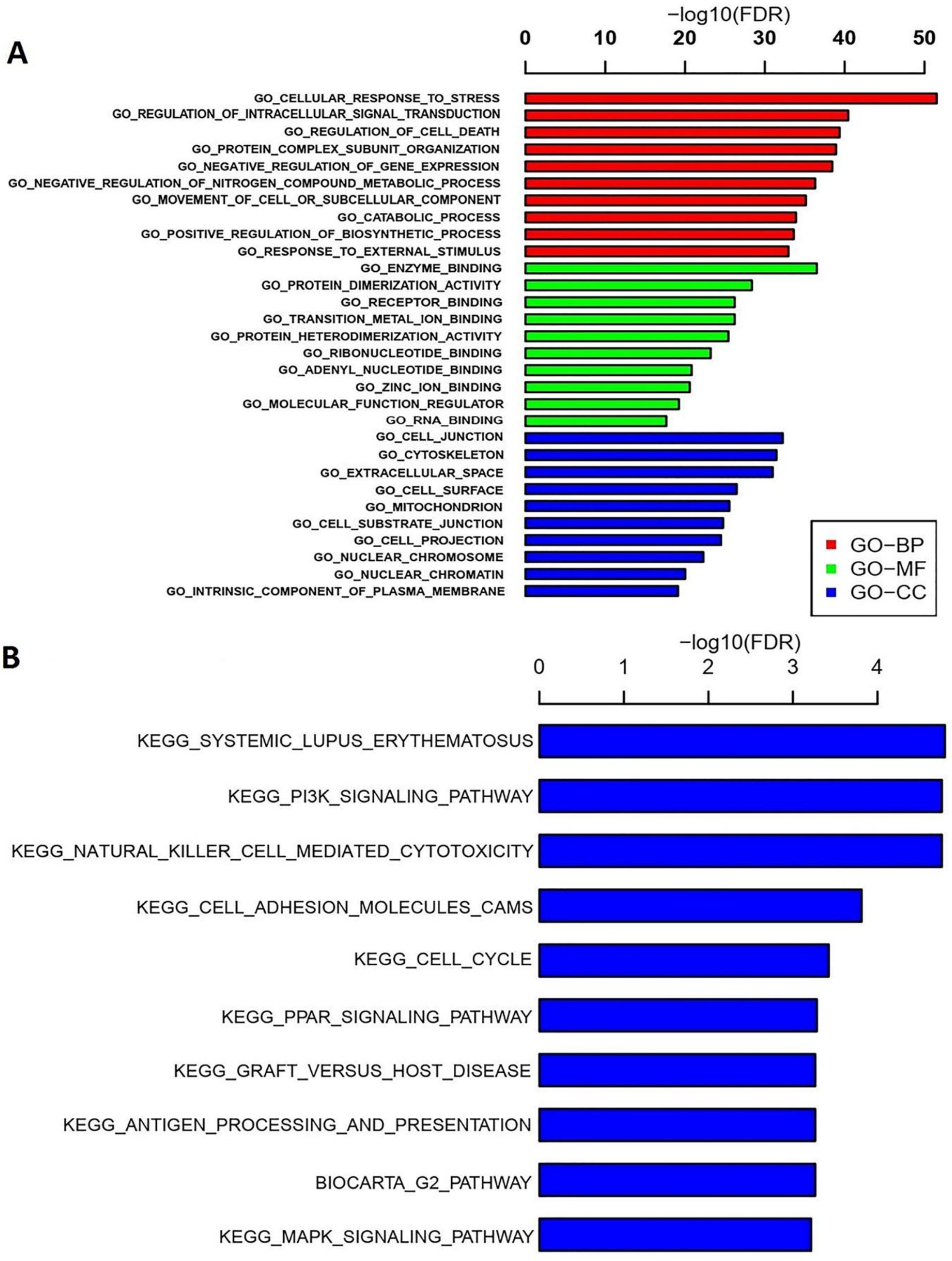

Supplementary Figure 6. Gene function and pathway enrichment of differentially expressed genes (DEGs) were determined by GO and KEGG analysis. (**A**) GO analysis was performed to evaluate the Biological Process (BP), Molecular Function (MF), and Cellular Component (CC) of these DEGs in Ubenimex- treated MKN-45/DDP cells, as described as the enrichment map of GO terms. (**B**) BioCarta or KEGG pathway analysis were carried out to clarify the pathway enrichment in these DEGs, as shown as the enrichment map of the top 10 over-represented canonical pathways following Ubenimex treatment.

Gene Set Name	Gene Symbol	Description	Fold Change
KEGG_PI3K_SIGNALING_PATH	NFKBIB	NFKB Inhibitor , beta	1.344551077
KEGG_PI3K_SIGNALING_PATH	NKRF	NFKB repressing factor	8.406317901
KEGG_PI3K_SIGNALING_PATH	PIK3R1	phosphoinositide-3-Kinase Regulatory Subunit 1	-12.908943940
KEGG_PI3K_SIGNALING_PATH	PIK3C2B	phosphatidylinositol-4-phosphate 3-kinase, catalytic subunit type 2 beta	-1.384418211
KEGG_PI3K_SIGNALING_PATH	PIK3R3	phosphoinositide-3-kinase, regulatory subunit 3 (gamma)	-1.181440701
KEGG_PI3K_SIGNALING_PATH	PIK3CB	phosphatidylinositol-4,5-bisphosphate 3-kinase, catalytic subunit beta	-1.116603143
KEGG_PI3K_SIGNALING_PATH	PIK3CG	phosphatidylinositol-4,5-bisphosphate 3-kinase, catalytic subunit gamma	-1.382075605
KEGG_PI3K_SIGNALING_PATH	PIK3C2A	phosphatidylinositol-4-phosphate 3-kinase, catalytic subunit type 2 alpha	-1.176025937
KEGG_PI3K_SIGNALING_PATH	PIK3CD	phosphatidylinositol-4,5-bisphosphate 3-kinase, catalytic subunit delta	-1.012538491
KEGG_PI3K_SIGNALING_PATH	PIK3CA	phosphatidylinositol-4,5-bisphosphate 3-kinase, catalytic subunit alpha	-1.416046764
KEGG_PI3K_SIGNALING_PATH	PIK3R5	phosphoinositide-3-kinase, regulatory subunit 5	-1.259684137
KEGG_PI3K_SIGNALING_PATH	PIK3IP1	phosphoinositide-3-kinase interacting protein 1	1.475801979
KEGG_PI3K_SIGNALING_PATH	PIK3C2G	phosphatidylinositol-4-phosphate 3-kinase, catalytic subunit type 2 gamma	-1.206000268
KEGG_PI3K_SIGNALING_PATH	PIK3R6	phosphoinositide-3-kinase, regulatory subunit 6	-1.107038243
KEGG_PI3K_SIGNALING_PATH	PIK3AP1	phosphoinositide-3-kinase adaptor protein 1	-1.170605016
KEGG_PI3K_SIGNALING_PATH	PIK3R4	phosphoinositide-3-kinase, regulatory subunit 4	-1.245926519
KEGG_PI3K_SIGNALING_PATH	NFKB2	nuclear factor of kappa light polypeptide gene enhancer in B-cells 2 (p49/p100)	-1.189951472
KEGG_PI3K_SIGNALING_PATH	NKAP	NFKB activating protein	-4.987823698
KEGG_PI3K_SIGNALING_PATH	NKIRAS2	NFKB inhibitor interacting Ras-like 2	1.383398614
KEGG_PI3K_SIGNALING_PATH	NKAPL	NFKB activating protein-like	-1.392148168
KEGG_PI3K_SIGNALING_PATH	NFKBIA	NFKB Inhibitor Alpha	9.558331407
KEGG_PI3K_SIGNALING_PATH	NFKB1	nuclear factor of kappa light polypeptide gene enhancer in B-cells 1	-1.810258653
KEGG_PI3K_SIGNALING_PATH	RELA	nuclear factor NF-kappa-B p65 subuni,t Transcription factor p65	-10.434542180
KEGG_PI3K_SIGNALING_PATH	NFKBIE	nuclear factor of kappa light polypeptide gene enhancer in B-cells inhibitor, epsilon	1.347048682
KEGG_PI3K_SIGNALING_PATH	NFKBID	nuclear factor of kappa light polypeptide gene enhancer in B-cells inhibitor, delta	1.382075605
KEGG_PI3K_SIGNALING_PATH	MTOR	mechanistic target of rapamycin (serine/threonine kinase)	-1.797650315
KEGG_PI3K_SIGNALING_PATH	GSKIP	GSK3B interacting protein	-1.848404711
KEGG_PI3K_SIGNALING_PATH	GSK3A	glycogen synthase kinase 3 alpha	1.101603517
KEGG_PI3K_SIGNALING_PATH	GSK3B	glycogen synthase kinase 3 beta	2.267654591
KEGG_PI3K_SIGNALING_PATH	IKBKB	inhibitor of kappa light polypeptide gene enhancer in B-cells, kinase beta	-1.609856482
KEGG_PI3K_SIGNALING_PATH	IKBKG	inhibitor of kappa light polypeptide gene enhancer in B-cells, kinase gamma	-1.739533094
KEGG_PI3K_SIGNALING_PATH	IKBKE	inhibitor of kappa light polypeptide gene enhancer in B-cells, kinase epsilon	-1.895365326
KEGG_PI3K_SIGNALING_PATH	AKT1	RAC-alpha serine/threonine-protein kinase	-11.554549540
KEGG_PI3K_SIGNALING_PATH	AKT2	AKT serine/threonine kinase 2;PKB beta	-6.545451724
KEGG_PI3K_SIGNALING_PATH	AKT3	AKT serine/threonine kinase 3;PKB gamma	-1.232389998
KEGG_PI3K_SIGNALING_PATH	AKTIP	AKT interacting protein	-1.257153923

Supplementary Figure 7. Details for the differentially expressed genes that were enriched in PI3Ksignaling pathway.
