# Supplementary figures and images for: Ubenimex induces autophagy inhibition and EMT suppression to overcome cisplatin resistance in GC cells by perturbing the CD13/EMP3/PI3K/AKT/NF-κB axis
Source: Aging (Albany NY). 2019 Dec 31;12(1):80–105. doi: 10.18632/aging.102598 (PMC6977684; doi:10.18632/aging.102598)

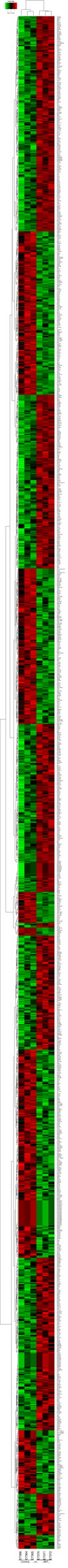

Supplement: Supplementary Database 1 [file aging-12-102598-s002..pdf]
